# The burden of hand trauma surgery on primary care in the United Kingdom: a nation-wide analysis of antibiotic and opioid prescriptions

**DOI:** 10.1177/17531934251338120

**Published:** 2025-05-08

**Authors:** Justin CR Wormald, Jennifer CE Lane, Marti Català, Antonella Delmestri, Jonathan Cook, Jeremy N Rodrigues, Matthew L Costa, Daniel Prieto-Alhambra

**Affiliations:** 1 Kadoorie Centre for Critical Care Research and Education, Nuffield Department of Orthopaedics, Rheumatology and Musculoskeletal Sciences, University of Oxford, UK; 2 Barts Bone and Joint Health, Faculty of Medicine and Dentistry, Queen Mary University of London, UK; 3 Botnar Institute for Musculoskeletal Sciences, Nuffield Department of Orthopaedics, Rheumatology and Musculoskeletal Sciences, University of Oxford, UK; 4 Warwick Clinical Trials Unit, Warwick Medical School, University of Warwick, UK; 5 Department of Plastic Surgery, Stoke Mandeville Hospital, Buckinghamshire Healthcare NHS Trust, UK; 6 Oxford Trauma and Emergency Care, Kadoorie Centre, Nuffield Department of Orthopaedics, Rheumatology and Musculoskeletal Sciences, University of Oxford, UK

**Keywords:** Antibiotics, hand surgery, infection, trauma

## Abstract

Although surgical site infection (SSI) risk after hand trauma surgery is around 5%, the severity of these infections is not known. The risk of superficial SSI in a cohort study was evaluated using NHS UK-wide primary care records (*n* = 641,223), using the Clinical Practice Research Datalink GOLD database. Within this cohort, a subcohort of those who had undergone a hand surgery operation for trauma were identified (*n* = 3,088). Antibiotic and analgesic prescriptions were analysed at 30 and 90 days postoperatively. By 30 days, 6.2% had been prescribed antibiotics appropriate for SSI, rising to 14.4% (CI [13.2 to 15.8]) by 90 days. By 30 days, 10% had been prescribed opioid analgaesia and by 90 days this had increased to 13.8%. Antibiotics prescriptions for SSI in primary care are substantially higher than the NICE estimate for SSI overall and the expected risk in hand trauma. The implications of this study are that many patients are receiving treatment for SSI in primary care and may be in more pain, for longer, than we expect. Further exploration of this is warranted and future research in hand trauma surgery should capture adverse events occurring outside of the hospital environment.

**Level of evidence:** II

## Introduction

Injuries of the hand and wrist, known collectively as ‘hand trauma’, are common and are increasing year on year (Manley et al., 2019). Data from NHS England revealed that in 2015/2016 there were 4.6 million patients who presented to the Emergency Department with hand trauma. Over 900,000, or one-in-five patients with hand trauma, required specialist care and 240,000 required surgery (Warwick, 2018). The majority of injuries occur in the young, working population (24–64 years old) and men are disproportionately affected (57%). The resulting disability causes loss of earnings, especially for those people who rely on their hands for work (Dias et al., 2020; Dias and Garcia-Elias, 2006).

The overall risk of surgical site infection (SSI) following hand trauma surgery is likely to be approximately 5.0–10%, based on a recent meta-analysis (Wormald et al., 2023). The type or severity of the SSIs that are counted within this figure remains unknown. The Centers for Disease Control and Prevention (CDC) define superficial SSI as occurring within 30 days (or 90 days if a surgical implant is used) and involving only skin and subcutaneous tissue of the incision (CDC, 2023). Many SSIs in the hand and wrist may be superficial, without systemic morbidity, and therefore patients may prefer to present to their General Practitioner rather than return to hospital (Warwick, 2018). Unlike in other musculoskeletal surgery, such as hip fracture or knee arthroplasty, the proportion of patients presenting to primary care is likely to be relatively high owing to the ‘day-case’ nature of most hand surgery (Warwick, 2018). This holds true for prolonged postoperative pain, although the risk of this is relatively unclear in hand trauma. This study aimed to explore whether there is a contingency of hand trauma surgery patients who solely present to primary care with SSI and require antibiotic treatment. The study secondarily evaluates postoperative analgesia requirements and wound care appointment data, further assessing the burden of hand trauma surgery on primary care services.

## Methods

### Study design

An observational study design to investigate the treatment of postoperative infection following surgery for hand trauma within primary care. Data derived from the Clinical Research Practice Datalink (CPRD) GOLD, mapped to the Observational Medical Outcomes Partnership (OMOP) Common Data Model (CDM) was used, comprising deidentified primary care data of 21 million patients. CPRD GOLD is representative of the UK population in terms of age, sex, ethnicity and BMI. The study is reported in accordance with the STROBE checklist (STROBE, 2023). All analyses were conducted within the ATLAS platform (OHDSI, 2024).

### Hand trauma cohort

A Read Code list was developed that generated a cohort of patients who had sustained injuries to the hand and/or wrist (Supplementary Material 1a). Patients were eligible for entry into the ‘hand trauma cohort’ if they had a relevant condition Read code within their medical record that indicated an injury to the hand or wrist. The hand trauma cohort includes patients who have sustained open and/or closed hand/wrist fractures, dislocations and subluxations, all soft-tissue injuries to the hand and wrist, including 465 specific Read codes for nerve, tendon, vessel and ligament injuries.

### Hand trauma surgery cohort

A second Read code list was then developed, consisting of procedural Read codes that relate to operations for a hand/wrist injury, including soft tissue closure, reconstruction of tendons and nerves, removal of foreign bodies and fracture and joint manipulation and fixation (Supplementary Material 1b). Patients were eligible to enter the ‘hand trauma surgery’ cohort if they underwent a relevant operative procedure within 10 days of injury. This cohort was generated by applying this criterion to the existing ‘hand trauma cohort’, so that only those who definitely underwent surgery could be analysed. This surgical cohort was defined using 159 Read codes, of which 67 pertained to soft tissue reconstruction (30 tendon repair, 14 soft tissue reconstruction, 8 amputation/terminalization and 15 other) and 93 pertained to fracture fixation (44 open reductions, 33 closed reductions and 16 other). Prior to analysis, cohort diagnostics were run to ensure that both code lists were comprehensive and included all relevant codes pertaining to hand trauma surgery. Cohort diagnostics was then used to identify potentially missing codes via in-built machine learning algorithms (OHDSI, 2024; Ostropolets et al., 2023; PHOEBE 2.0, 2022) Missing codes were reviewed and included if relevant. For both cohorts, characterization analyses were performed to determine population demographics (age and sex), injury types and prevalence, and the types and frequency of surgical procedures.

### Outcome measurements

Prescription data were extracted for up to 30 days and up to 90 days to ascertain treatment with antibiotics in these time periods following surgery (Supplementary Material 2). Prescription of antibiotics in the specified time periods is a valid proxy for postoperative infection based on the existing literature (Daryapeyma et al., 2016; Hartmann et al., 2004; Kruse et al., 2006; Stridh Ekman et al., 2010; Whitby et al., 2002). This method was further validated for this study by refining the type of antibiotic prescribed compared with pre-injury prescription rates and only analysing those potentially relevant to superficial wound infection. As these are solely oral antibiotic prescriptions, one can infer that the type of SSI that is being treated is superficial SSI. Deep or joint space SSIs would require admission for intravenous antibiotics and/or surgery, whereas superficial SSI would be managed with oral antibiotics alone in the majority of cases (CDC, 2023). The number of incident prescriptions was used as a surrogate for new cases of superficial SSI by 30 days and by 90 days. First, all antibiotic prescriptions were quantified. Prescription data for the most commonly used antibiotics in skin and soft-tissue infections were then extracted. Flucloxacillin (floxacillin) prescription alone was then further analysed, with both pre- and postoperative prescription rates checked to determine clinically meaningful change in its prescription. Flucloxacillin is commonly prescribed in primary care for superficial SSI, as *Staphylococcus aureus* is the most common pathogenic organism (McDonald et al., 2011; Oxford Health NHS Foundation Trust Formulary, 2024). The prescription data are cumulative, such that the ‘by 90 days’ data will also include the ‘by 30 days’ data. Prescription data prior to either hand injury (hand trauma cohort) or hand trauma surgery (hand trauma surgery cohort) were used to verify increased postoperative prescription rates.

#### Postoperative pain

Prescription of analgesia following surgery and follow-up data ostensibly pertaining to wound care were secondarily explored. Prescription data for analgesics were similarly extracted for up to 30 days and up to 90 days following hand trauma surgery. Analgesics were chosen based on the most commonly prescribed agents in clinical practice for day case surgery, combining non-steroidal anti-inflammatory drugs (NSAIDs) and opioid agents. The NSAIDs were ibuprofen, diclofenac and naproxen and the opioids were codeine, dihydrocodeine and tramadol.

#### Utilization of primary care

A third objective was to assess the most common reasons why hand trauma patients presented to primary care services following surgery. The codes used to classify and describe primary care appointments at 30 and 90 days following injury were explored for the hand trauma cohort and following surgery for the hand trauma cohort surgery.

### Data source

CPRD GOLD is a data source containing national electronic healthcare data, is based in primary care and holds anonymized electronic health record data from collaborating general practices, with all patients registered at those practices included in the dataset, unless they have opted out (CPRD, 2022). CPRD GOLD July 2020 was used for this study, with the final analysis in January 2023 (CPRD GOLD, 2023).

### Risk of bias

CPRD GOLD is validated and quality checked before data are released for analysis. This reduces missingness and biases that may arise as a result. National coverage is not complete as only certain practices may contribute data, subject to process and quality control (Herrett et al., 2015). However, the data contained within CPRD GOLD are representative of the UK population. Misclassification of injuries and procedures is a potential area for bias, as Read codes may be attributed to patient records by those not familiar with the speciality area. This was mitigated through cohort diagnostics, allowing clinical validation of the included definitions. A snapshot of metadata for the included population was reviewed and potential missing Read codes were suggested/added.

### Sample size and statistical analyses

This is a descriptive study and therefore a formal sample size calculation was not necessary. Descriptive analyses are used to summarize demographic, treatment and prescription data. Sex was analysed as proportion of males and females (%) with 95% confidence intervals (CIs). Age was presented as a mean and standard deviation (SD). Prescription data for antibiotics, NSAIDs and opioids were analysed as a proportion (%) of those that had been prescribed each drug out of the total cohorts: hand trauma and hand trauma surgery. All 95% CIs were calculated using binomial exact calculation using Microsoft Excel version 16.71 (Stephanie, 2018).

## Results

The CPRD GOLD July 2020 source population includes 19,018,786 patient records, of which 3,185,210 are currently registered as of January 2023 when this analysis was performed. This represents 4.8% of the UK population.

## Hand trauma cohort

**Figure 1.**
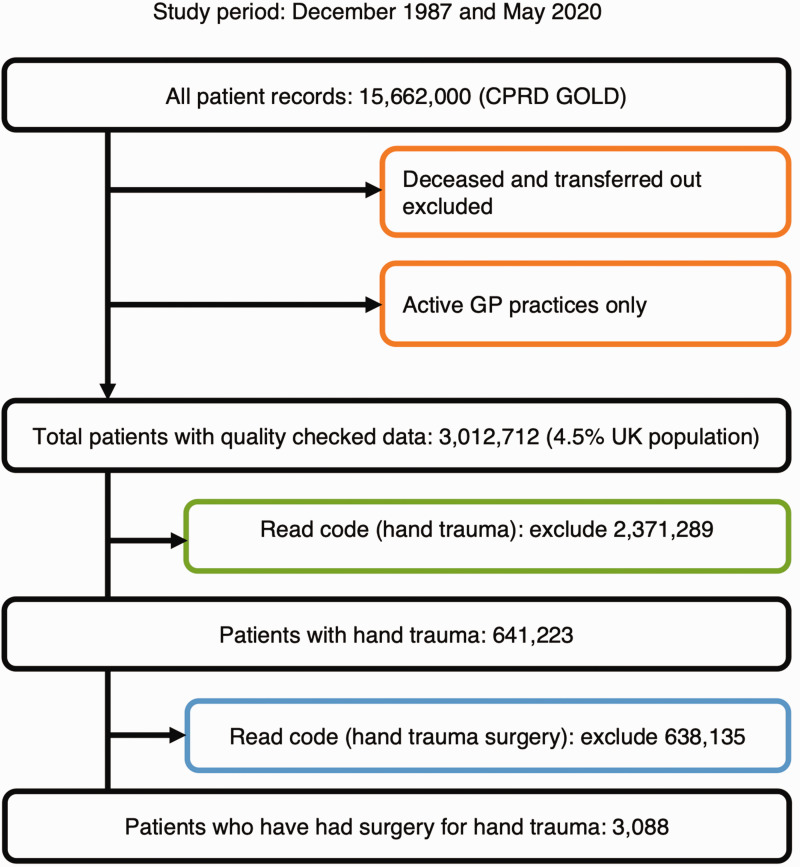
Flow chart of study attrition.

**Figure 2.**
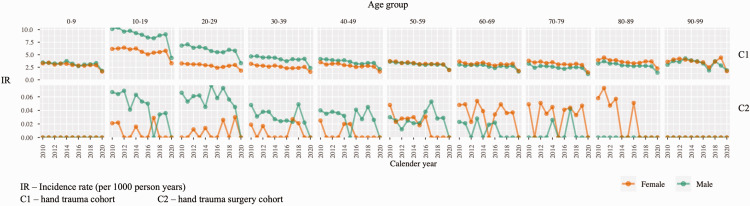
Demographic data for Clinical Practice Research Datalink (CPRD) cohorts.

This section presents data on all patients who sustained hand trauma, regardless of whether they underwent surgery. Using the ‘hand trauma’ Read code list, records from 641,223 patients were extracted to form this cohort (Supplementary Material 1a, Figure 1). The cohort included a diverse mix of individuals, with a higher proportion of males compared with females (Figure 2). The cohort’s average age was within a range typically associated with general health, and most individuals had few or no comorbidities, with the majority of conditions affecting less than 1% of the population.

### Surgical site infection

A wide range of antibiotics were prescribed within this cohort. Notably, the antibiotics were not categorized specifically for SSI, as some patients did not have surgery. Within the first 30 days, 6.2% of patients had been prescribed antibiotics, and this number increased to 15.5% over a 90 day period. When focusing on antibiotics typically used for skin and wound infections, 5.2% of patients received prescriptions within 30 days, with the number continuing to rise to 13.9% by 90 days. Flucloxacillin was used in 1.2% of patients within the 30 day period and continued to be prescribed in 2.4% of patients within the 90 day window. Similarly, amoxicillin was also prescribed more frequently over time (Table 1).

**Table 1.** Prescription of antibiotics, non-steroidal anti-inflammatory drugs and opioids at 30 and 90 Days post-injury (*n* = 641,223)

Medication	30 Days (*n*, % [95% CI])	90 Days (*n*, % [95% CI])
*Antibiotics*		
Flucloxacillin	7,429 (1.2%)	15,094 (2.4%)
Amoxicillin	11,392 (1.8%)	33,160 (5.2%)
Clavulanate	3,061 (0.5%)	6,668 (1.0%)
Fusidate	2,795 (0.4%)	8,012 (1.2%)
Erythromycin	3,723 (0.6%)	10,008 (1.6%)
Clarithromycin	1,327 (0.2%)	3,706 (0.6%)
Clindamycin	461 (0.1%)	1,401 (0.2%)
Doxycycline	1,120 (0.2%)	3,630 (0.6%)
Ciprofloxacin	805 (0.1%)	2,289 (0.4%)
Chloramphenicol	1,683 (0.3%)	5,333 (0.8%)
***Total antibiotics***	**33,796 (5.2% [5.2 to 5.3])**	**89,301 (13.9% [13.8 to 14.0])**
*NSAIDs*		
Ibuprofen	6,473 (1.0%)	15,920 (2.5%)
Diclofenac	4,840 (0.8%)	12,093 (1.9%)
Naproxen	2,317 (0.4%)	5,912 (0.9%)
***Total NSAIDs***	**13,630 (2.1% [2.1 to 2.2])**	**33,925 (5.3% [5.2 to 5.4])**
*Opioids*		
Codeine	10,154 (1.6%)	20,981 (3.3%)
Dihydrocodeine	3,752 (0.6%)	7,864 (1.2%)
Tramadol	2,015 (0.3%)	4,415 (0.7%)
***Total opioids***	**15,921 (2.5% [2.4 to 2.5])**	**33,260 (5.2% [5.1 to 5.2])**

Note: Percentages represent the proportion of patients prescribed each medication category within 30 and 90 days post-injury. Confidence intervals (CIs) are provided in brackets.

### Postoperative pain management

The use of NSAIDs was rare within the cohort, with prescription rates remaining consistently low and showing no significant increase following hand and wrist injuries. In contrast, opioid analgesics were more commonly prescribed, with a 2.5% of patients receiving them within the 30 day period post-injury, and this figure increased to 5.2% within the 90 day period.

### Utilization of primary care

During the first 30 days following their injury, patients most commonly sought follow-up care for wound dressings (2.8%), clinical reviews (2.7%) and suture removal (1.2%).

#### Hand trauma surgery cohort

A more specific subset of 3088 patients was identified from the larger cohort, all of whom had undergone surgery for hand or wrist injuries (Supplementary Material 1b, Figure 1). This surgical group had a higher proportion of males and was also generally healthy, with a lower incidence of comorbidities compared to the larger hand trauma group.

#### Surgical site infection

All patients in this subgroup had undergone surgery, meaning antibiotic prescriptions were more directly related to the treatment of SSI. Antibiotic use was relatively common, particularly for wound and skin infections. Antibiotics specifically used for wound and skin infections were prescribed for 6.2% of patients by 30 days and 14.5% by 90 days. Flucloxacillin was frequently prescribed within the 30 day and 90 day periods (2.2 and 4.5% respectively), with a significant increase from pre-injury prescription rates (*χ*^2^ = 11.5, *p* < 0.005). Similarly, amoxicillin use also rose significantly post-surgery (Table 2).

**Table 2.** Antibiotic, non-steroidal anti-inflammatory drug and opioid prescription following hand trauma surgery (*n* = 3088)

Medication	30 Days (*n*, % [95% CI])	90 Days (*n*, % [95% CI])
*Antibiotics*		
Flucloxacillin	68 (2.2%)	140 (4.5%)
Amoxicillin	46 (1.5%)	127 (4.1%)
Clavulanate	26 (0.8%)	41 (1.3%)
Fusidate	10 (0.3%)	31 (1.0%)
Erythromycin	12 (0.4%)	31 (1.0%)
Clarithromycin	10 (0.3%)	26 (0.8%)
Clindamycin	5 (0.2%)	8 (0.3%)
Doxycycline	5 (0.2%)	15 (0.5%)
Ciprofloxacin	4 (0.1%)	10 (0.3%)
Chloramphenicol	6 (0.2%)	18 (0.6%)
***Total antibiotics***	**192 (6.2% [5.4 to 7.1])**	**447 (14.4% [13.2 to 15.8])**
*NSAIDs*		
Ibuprofen	59 (1.9%)	110 (3.6%)
Diclofenac	54 (1.7%)	81 (2.6%)
Naproxen	17 (0.6%)	46 (1.5%)
***Total NSAIDs***	**130 (4.2% [3.5 to 5.0])**	**237 (7.7% [6.8 to 8.7])**
*Opioids*		
Codeine	186 (6.0%)	255 (8.3%)
Dihydrocodeine	68 (2.2%)	89 (2.9%)
Tramadol	54 (1.7%)	81 (2.6%)
*total opioids*	**308 (10.0% [8.9–11.1])**	**425 (13.8% [12.6–15.0])**

Note: Percentages represent the proportion of patients prescribed each medication category within 30 and 90 days post-surgery. Confidence intervals (CIs) are provided in brackets.

### Postoperative pain management

Non-steroidal anti-inflammatory drug prescriptions increased in the surgical cohort compared to the general hand trauma group, with a notable rise in prescriptions following surgery. Opioid prescriptions were also more frequent, with 10.0% of post-surgical patients receiving them within 30 days. By 90 days, this increased to 13.8%.

### Utilization of primary care

Patients in the surgical cohort required more follow-up care compared with the broader hand trauma group. Within 30 days of surgery, 4.4% of patients required wound dressing changes, suture removal was needed in 3.4% and removal of K-wires was required in 1.7% within 30 days. These all reduced by 90 days, other than K-wire removal, which increased 2.4%.

## Discussion

This is a national study of 3088 patients who have undergone surgery for hand and wrist trauma and have subsequently attended primary care with a postoperative complication. Using the prescription of clinically appropriate antibiotics within 30 and 90 days after surgery as a proxy for SSI, the risk of SSI by 30 days is 6.2%. This rises further to 14.4% by 90 days. Opioid prescriptions were higher than anticipated, raising new concerns regarding chronic pain and opioid dependence. Less than 5% of the surgery cohort attended primary care for wound dressings or suture removal. Data from hand trauma studies during and since the COVID-19 pandemic demonstrate that most postoperative hand trauma wound reviews and suture removals are performed in hospital, in some part explaining the low utilization seen here (Shaw et al., 2023; Shafi et al., 2024).

The overall risk of SSI following hand trauma surgery is approximately 5%, based on a meta-analysis of 315,618 patients who had undergone surgery for hand and wrist trauma (Wormald et al., 2023). The risk of SSI was higher in prospective RCTs (10.1%) (Wormald et al., 2023). This meta-analysis did not contain any studies based in primary care. This present study adds granularity through analysis of primary care data to produce risk estimates for superficial SSI following hand trauma surgery. The SSI risks generated through this CPRD GOLD analysis sit on the upper end of the spectrum of SSI risk and are far more common than the National Institute for Health and Care Excellence (NICE) estimate of 2–5% (NICE, 2019). The increase in antibiotic prescription at 90 days is unexpected and may represent a true increase in SSIs or an inappropriate/over-prescription of antibiotics. By 90 days, a surgical wound will have formed a scar and will be maturing, becoming more erythematous as angiogenesis takes place (Grubbs and Manna, 2025). This could be misinterpreted as nascent infection, which could result in overdiagnosis of superficial SSI.

Studies of musculoskeletal trauma within CPRD GOLD generally appear to have higher SSI incidences compared with those in other studies of national databases. This is the case for both orthopaedic trauma (3.5%, clavicular fixation, 11.7% tibial fixation) and elective arthroplasty (4.1% total knee replacement) (Galvain et al., 2020; Wolf et al., 2022; Wallace et al., 2014). In contrast, the figures from the National Joint Registry, a secondary care dataset, of total knee replacement outcomes found that postoperative infection was the reason for revision surgery in 412 out of 341,749 operations, equating to a deep infection risk of 0.1% (Liddle et al., 2014). This demonstrates that secondary care datasets detect the most serious SSI events but may underestimate the overall SSI risk for a population or procedure. Alternatively, the higher treatment rates seen in CPRD may reflect overprescribing of antibiotics, either for prophylaxis or over-treatment of non-infections. Overprescribing is known to be an issue in primary care and is a major concern regarding antibiotic resistance (Gulliford et al., 2020; Krockow et al., 2022; Llor and Bjerrum, 2014). Importantly, there is scant published evidence that General Practitioners diagnose and treat SSI incorrectly. Indeed, there is data suggesting that patients’ self-diagnosis of SSI can be at least as accurate as a surgeon’s diagnosis (Pham et al., 2016; Whitby et al., 2002).

In the existing literature, the majority of hand trauma SSIs that present to the hospital are managed successfully with oral antibiotics alone. In Pavan et al.’s (2018) retrospective series of 638 hand trauma patients, 19 (3%) developed SSI, 14 requiring oral antibiotics alone. In Davies et al.’s (2020) series of 983 hand trauma patients, 41 developed SSI (4.2%), with 33 receiving oral antibiotics alone. In Baldwin et al.’s (2021) series of 556 hand trauma patients, 20 (3.6%) developed SSI and 8 had antibiotics only. If the majority of hand trauma SSIs are treated with oral antibiotics only, then it is logical that many will exclusively be managed in primary care. This study confirms this theory, demonstrating high rates of antibiotic prescription in the community following hand trauma surgery.

It is known that codeine and tramadol are deleterious to health and well-being, with the latter being particularly harmful in certain populations (Xie et al., 2021). A high prevalence of opioid analgesic prescriptions has been found in the surgical cohort in this study. In the USA, overprescription of opioid analgesia has resulted in a health crisis, and prescription of opioids after minor surgical procedures has contributed to this (Stoicea et al., 2019). There is evidence that a proportion of patients continue to use opioid analgesia over 90 days after simple elective hand surgical procedures in the USA (Johnson et al., 2016). There is further data from the USA exploring reasons for prolonged opioid use following non-hand musculoskeletal surgery (Helmerhorst et al., 2014). As such, the hand surgery communities in the USA and Canada are attempting to reduce opioid use by generating evidence for alternative analgesic strategies (Lalonde et al., 2022). It is concerning that these new data show a similar pattern for hand trauma patients in the UK.

There are limitations in this study. This analysis provides new results indicating that a significant proportion of patients will present to their General Practitioner requiring antibiotic treatment that is consistent with soft tissue infection following surgery for hand trauma. The logical conclusion is that these patients are presenting with SSI. However, there may be patients who have co-morbid skin infections. These results should therefore be interpreted as a signal, with antibiotic prescription as a surrogate for SSI, rather than a conclusive finding. There will also be an unknown proportion of patients who are prescribed antibiotics for a wound that is inflamed rather than infected, i.e. suspected SSI.

Assessment of SSI following hand trauma surgery should take into account its manifestation in both primary and secondary care. The primary care physician is faced with a higher than previously anticipated burden following hand trauma surgery, in terms of both postoperative infection and pain. Hand surgeons should consider these findings in the context of developing postoperative pathways that alleviate the clinical burden from non-specialists who may be ill-equipped to navigate the postoperative course of hand trauma patients.

## Supplemental Material

sj-pdf-1-jhs-10.1177_17531934251338120 - Supplemental material for The burden of hand trauma surgery on primary care in the United Kingdom: a nation-wide analysis of antibiotic and opioid prescriptions Supplemental material, sj-pdf-1-jhs-10.1177_17531934251338120 for The burden of hand trauma surgery on primary care in the United Kingdom: a nation-wide analysis of antibiotic and opioid prescriptions by Justin CR Wormald, Jennifer CE Lane, Marti Català, Antonella Delmestri, Jonathan Cook, Jeremy N Rodrigues, Matthew L Costa and Daniel Prieto-Alhambra in Journal of Hand Surgery (European Volume)
