# Supplementary material for: The burden of hand trauma surgery on primary care in the United Kingdom: a nation-wide analysis of antibiotic and opioid prescriptions
Source: J Hand Surg Eur Vol. 2025 May 8;50(10):1356–64. doi: 10.1177/17531934251338120 (PMC12559369; doi:10.1177/17531934251338120)
Supplement: sj-pdf-1-jhs-10.1177_17531934251338120 - Supplemental material for The burden of hand trauma surgery on primary care in the United Kingdom: a nation-wide analysis of antibiotic and opioid prescriptions [file sj-pdf-1-jhs-10.1177_17531934251338120.pdf]

Supplementary Material 1a. Read codes: hand trauma cohort

Concept	Concept name
4051298	Traumatic amputation, through wrist
45500683	Closed traumatic dislocation other carpal joint
45424151	Open division wrist ligament NOS
45493795	Other symptoms in proximal interphalangeal joint of finger
4062229	Closed injury, common digital nerve
45427435	Traumatic amputation, through wrist
45430913	[X]Traumatic amputation of two or more fingers alone (complete)(partial)
45490815	Injury of unspecified blood vessel at wrist and hand level
45494225	Closed injury, common digital nerve
45480885	Other closed traumatic dislocation of wrist
45471090	[X]Crushing injury of other and unspecified parts of wrist and hand
4062230	Open injury, common digital nerve
45430775	Traumatic amputation of arm and hand NOS
4050550	Open injury, digital artery
45510771	Injury of unspecified muscle and tendon at wrist and hand level
45497490	Injury of extensor muscle and tendon of other finger(s) at forearm level
45514134	Open injury, digital artery
45520775	Open injury, common digital nerve
45474180	Open division finger ligament NOS
4052992	Degloving injury hand, dorsum
45447334	Closed injury, digital vein
4050380	Degloving injury, finger, multiple
45454027	Superficial injury of wrist NOS, infected
45480886	Closed traumatic subluxation of the wrist, unspecified
45430772	Degloving injury hand, dorsum
4055589	Closed injury of digital vein
45460696	Degloving injury, finger, multiple
45457343	Hand dislocation NOS

45484141	Open fracture of middle or proximal phalanx or phalanges, unspecified part
45424148	Complete tear ligament finger NOS
45510760	Open injury, digital nerve, multiple
45427369	Open traumatic dislocation of finger, unspecified
45464143	Complete tear wrist or hand NOS
4059101	Open injury, digital nerve, multiple
45497539	[X]Injury of unspecified nerve at wrist and hand level
45504140	Injury of flexor muscle and tendon of other finger(s) at forearm level
45437313	Open fracture lunate
45427430	Partial division flexor tendon wrist
45490798	Partial division flexor tendon hand
4051154	Partial division flexor tendon wrist
45487471	Open fracture trapezium
442765	Open fracture of trapezium of wrist
45443931	Open fracture finger middle phalanx, base
73616	Open fracture of lunate bone of wrist
45430734	Closed traumatic dislocation of wrist not otherwise specified
4015489	Open fracture finger middle phalanx, base
4050229	Partial division flexor tendon hand
45500812	Closed injury, digital nerve in finger
4057344	Contusion wrist, volar
45440757	Closed crush injury wrist, dorsum
4057019	Closed injury, digital nerve in finger
45424191	Traumatic amputation of wrist and hand, level unspecified
45444015	Complete division, both flexor tendons
4051898	Closed crush injury wrist, dorsum
4052670	Contusion wrist, dorsum
45424222	Contusion wrist, volar
45470997	Contusion wrist, dorsum
45490803	Traumatic amputation, finger, through proximal interphalangeal joint
45474140	Open multiple fractures of hand bones
434184	Multiple open fractures of hand bones

4014518	Rupture wrist flexors
45460663	Rupture wrist flexors
4002799	Dislocation of tendon, wrist or hand
4053126	Traumatic amputation, finger, through proximal interphalangeal joint
45470921	Complete tear wrist ligament NOS
45470657	Dislocation of tendon, wrist or hand
4012304	Open fracture finger middle phalanx, shaft
45490814	Digital blood vessel injury
194209	Digital blood vessel injury
45514155	Open crush injury hand, palm
45477496	Closed fracture finger middle phalanx, multiple
4010532	Closed fracture finger middle phalanx, multiple
45424109	Open fracture finger middle phalanx, shaft
45453969	Complete tear wrist ligament
4059551	Open crush injury hand, palm
45500668	Open fracture finger distal phalanx, shaft
4056297	Traumatic amputation fingertip, type 2
4009450	Open fracture finger metacarpal base
45490804	Traumatic amputation finger tip, type 2 (pulp and nail loss)
4015965	Open fracture finger distal phalanx, shaft
45443929	Open fracture finger metacarpal base
4009594	Closed fracture finger distal phalanx, multiple
4057478	Closed crush injury hand, palm
45464107	Closed fracture finger distal phalanx, multiple
45470882	Open fracture finger metacarpal head
45514126	Traumatic amputation, finger, through distal interphalangeal joint
45430819	Closed crush injury hand, palm
4009451	Open fracture finger metacarpal head
79957	Traumatic amputation of finger with complication
4051294	Traumatic amputation, finger, through distal interphalangeal joint
4009597	Open fracture finger proximal phalanx, shaft
4050387	Traumatic amputation fingertip, type 1

45427433	Traumatic amputation of finger with complication
45517398	Open fracture finger proximal phalanx, shaft
45470966	Traumatic amputation finger tip, type 1 (pulp only involved)
4050222	Complete division flexor tendon wrist
45460637	Closed traumatic subluxation of the wrist
45487543	Complete division flexor tendon wrist
45514045	Open fracture triquetral
45494158	Degloving injury, finger unspecified
76838	Open fracture of triquetral bone of wrist
45523986	Open wound of fingernail
45510640	Open division wrist ligament
4059434	Open crush injury hand, dorsum
45470925	Rupture wrist extensors
45474333	[X]Contusion of other parts of wrist and hand
4014383	Open division wrist ligament
4016828	Rupture wrist extensors
45474249	Open crush injury hand, dorsum
45500701	Open fracture dislocation wrist
4015062	Open fracture dislocation wrist
438004	Open fracture of hamate bone of wrist
45524109	[X]Sprain and strain of other and unspecified parts of wrist and hand
45477490	Open fracture hamate
4012728	Closed traumatic subluxation of wrist
45447237	Wrist fracture - open
4019055	Complete tear of wrist ligament
45454026	Splinter of wrist, without major open wound
4058223	Splinter of wrist, without major open wound
45430754	Open division finger ligament
45440653	Closed traumatic dislocation of wrist
45490797	Complete division flexor tendon hand
45447242	Open fracture finger proximal phalanx, base
4015487	Open fracture finger proximal phalanx, base

4002798	Subluxation of tendon, wrist or hand
45520411	Subluxation of tendon, wrist or hand
45494157	Open wound of finger or thumb with complication
4012429	Open fracture finger distal phalanx, base
4014387	Open division finger ligament
4344504	Rupture of tendon of wrist and hand
45520688	Rupture tendon hand or wrist NOS
45464108	Open fracture finger distal phalanx, base
45430717	Closed fracture finger proximal phalanx, multiple
45447327	Traumatic amputation of arm and hand
4009461	Closed fracture finger proximal phalanx, multiple
45507408	Other elbow, forearm and wrist injuries
45523913	Closed fracture finger middle phalanx, neck
45494071	Multiple fractures of hand bones NOS
4010531	Closed fracture finger middle phalanx, neck
4056162	Complete division flexor tendon hand
45514063	Carpal dislocation NOS
4015483	Closed fracture finger middle phalanx, head
45453919	Closed fracture finger middle phalanx, head
45484216	Traumatic amputation of finger without mention of complication
4084891	Self-mutilation of hands
45448017	Self-mutilation of hands
45440681	Complete tear, wrist or hand
45490838	Splinter of hand, without major open wound or mention of infection
4015361	Open fracture finger metacarpal neck
4052209	Injury of ulnar artery at wrist and hand level
45510720	Open crush injury, finger, multiple
45427351	Open fracture finger metacarpal neck
4018968	Sprain finger, proximal interphalangeal joint, radial collateral ligament
4057479	Open crush injury, finger, multiple
443231	Traumatic amputation of arm and hand
45514092	Complete tear ligament finger

45497429	Injury of ulnar artery at wrist and hand level
45450628	Sprain finger, proximal interphalangeal joint, radial collateral ligament
45503981	Closed fracture finger metacarpal, multiple
4010387	Closed fracture finger metacarpal, multiple
4019054	Complete tear of ligament of wrist and/or hand
4018628	Complete tear of ligament of finger
45487541	Open wound of wrist, dorsal
4050220	Open wound of wrist, dorsal
434193	Closed traumatic dislocation of distal radioulnar joint of wrist
45464117	Closed traumatic dislocation distal radio-ulnar joint
45447321	Open wound of lower arm, NOS
4050234	Mallet finger with open tendon injury
45504060	Mallet finger with open tendon injury
72481	Open wound of wrist with complication
45504059	Open wound of wrist with complication
45430718	Closed fracture finger distal phalanx, shaft
4010533	Closed fracture finger distal phalanx, shaft
45504198	[X]Fracture of other and unspecified parts of wrist and hand
45510719	Closed crush injury hand, dorsum
4054859	Injury of radial nerve at wrist and hand level
45494224	Injury of radial nerve at wrist and hand level
4057477	Closed crush injury hand, dorsum
45420934	Rupture tendon of finger NOS
45474204	Complete division extensor tendon hand
4051282	Complete division extensor tendon hand
45490796	Multiple open wounds of wrist and hand
4191822	Multiple open wounds of wrist and hand
434773	Closed multiple fractures of hand bones
45440639	Closed multiple fractures of hand bones
45484144	Open fracture of one or more phalanges of hand NOS
45484136	Hand fracture - carpal bone
45497331	Open fracture of distal phalanx or phalanges, unspecified part

45450593	Open fracture of the scaphoid
77124	Open fracture of scaphoid bone of wrist
4059100	Open injury, digital nerve in finger
45520676	Sprain tendon wrist or hand
45427504	Open injury, digital nerve in finger
45490729	Multiple fractures of metacarpal bones
4009460	Closed fracture finger proximal phalanx, neck
45484140	Closed fracture finger proximal phalanx, neck
45427349	Closed fracture trapezoid
436551	Closed fracture of trapezoidal bone of wrist
45514086	Sprain wrist flexors
36683549	Strain of wrist flexor tendon
45420957	Open wound of wrist, volar
4052981	Open wound of wrist, volar
45517469	Partial division extensor tendon hand
4050228	Partial division extensor tendon hand
4059553	Closed crush injury, finger, multiple
45477513	Closed traumatic dislocation of wrist, unspecified
45517508	Closed crush injury, finger, multiple
45510602	Open fracture finger distal phalanx, tuft
4015491	Open fracture finger distal phalanx, tuft
45470999	Crush injury, wrist and hand NOS
36683548	Strain of wrist extensor tendon
45500710	Sprain wrist extensors
45457313	Closed fracture finger middle phalanx, shaft
4009592	Closed fracture finger middle phalanx, shaft
4054857	Injury of ulnar nerve at wrist and hand level
45450743	Injury of ulnar nerve at wrist and hand level
4015878	Closed fracture finger proximal phalanx, head
45457312	Closed fracture finger proximal phalanx, head
73906	Open wound of wrist with tendon involvement
45514123	Open wound of wrist with tendon involvement

4050232	Open wound, finger, multiple
45440709	Open wound, finger, multiple
45474205	Degloving injury hand
437406	Closed traumatic dislocation of joint of wrist
4052990	Degloving injury of hand
45464119	Dislocation of wrist NOS
4018225	Closed fracture subluxation of the wrist
45460649	Closed fracture-subluxation of the wrist
45437515	[X]Injuries to the wrist and hand
45460624	Open fracture of phalanx or phalanges, unspecified
45500667	Closed fracture finger distal phalanx, mallet
4010535	Closed fracture finger distal phalanx, mallet
45460626	Open fracture finger middle phalanx
73042	Open fracture finger middle phalanx
45477574	Open wound of finger with tendon injury
45487478	Multiple fractures of hand bones
443114	Open fracture finger proximal phalanx
45503984	Closed fracture finger proximal phalanx, shaft
45440638	Open fracture finger proximal phalanx
4010530	Closed fracture finger proximal phalanx, shaft
4015873	Open fracture finger metacarpal
45487476	Open fracture finger metacarpal
4292073	Multiple fractures of hand bones
80854	Closed fracture of capitate bone of wrist
45510597	Closed fracture capitate
45430770	Open wound of elbow, forearm and wrist NOS
45464214	Superficial injury of hand NOS, infected
4010534	Closed fracture finger distal phalanx, tuft
45520709	Open wound of lower arm without mention of complication
45497329	Closed fracture finger distal phalanx, tuft
4010527	Closed fracture of sesamoid bone of hand
45437315	Closed fracture sesamoid bone of hand

45444014	Open wound of hand with tendon involvement
4056161	Open wound of hand with tendon involvement
45504061	Degloving injury, finger
4003136	Hand and wrist flexor tendon rupture
45473900	Hand and wrist flexor tendon rupture
78274	Closed fracture of pisiform bone of wrist
45517396	Closed fracture pisiform
4052998	Degloving injury of finger
45504133	Injury of unspecified nerve at wrist and hand level
37110530	Injury of nerve at hand level
45477627	Injury of digital nerve of thumb
4062231	Injury of digital nerve of thumb
45464185	Traumatic amputation of finger NOS
45490728	Closed fracture lunate
75375	Closed fracture of lunate bone of wrist
45467574	Contusion, finger NOS
45464181	Open wound of finger or thumb with tendon involvement
4052524	Abrasion of finger
45510699	Abrasion, finger
45427354	Closed fracture of one or more phalanges of hand NOS
45484139	Closed fracture of middle or proximal phalanx or phalanges, unspecified part
45484217	Traumatic amputation, finger tip
4096480	Traumatic amputation of fingertip
45434010	Hand fracture - metacarpal bone
441977	Closed traumatic dislocation of carpometacarpal joint of wrist
45460635	Closed traumatic dislocation carpometacarpal joint
45484248	Contusion, wrist and hand NOS
45440708	Open wound of hand, dorsum
4052989	Open wound of hand, dorsum
45501029	Slashed wrists self inflicted
45444086	Tendon injury to hand NOS
45514062	Dislocation or subluxation of wrist

45450627	Hand sprain NOS
4009593	Closed fracture finger distal phalanx, base
45487477	Closed fracture finger distal phalanx, base
4054986	Open crush injury, finger
45484256	Open crush injury, finger
45477535	Closed fracture dislocation of wrist
4018221	Closed fracture dislocation of wrist
45487563	Superficial injury of finger NOS, infected
45490813	Injury of radial artery at wrist and hand level
4050547	Injury of radial artery at wrist and hand level
4168061	Open wound of finger with tendon injury
45440676	Finger sprain
45470883	Closed fracture of distal phalanx or phalanges, unspecified part
45470963	Open wound fingernail
45424142	Sprain tendon of finger
36683537	Strain of finger tendon
45514046	Closed fracture finger metacarpal head
4015355	Closed fracture finger metacarpal head
45447358	Crush injury wrist or hand
4003135	Hand and wrist extensor tendon rupture
45477253	Hand and wrist extensor tendon rupture
4053836	Open wound of fingernail
4016263	Dislocation or subluxation of wrist
45447346	Multiple superficial injuries of wrist and hand
4051597	Multiple superficial injuries of wrist and hand
45427432	Open wound of finger or thumb without mention of complication
45424139	Hand sprain unspecified
45447241	Open fracture of one or more phalanges of hand
4058513	Crush injury wrist and/or hand
433886	Closed fracture of trapezium bone of wrist
45480875	Closed fracture trapezium
45450664	Open wound of finger with damage to nail

44789002	Open wound of finger with damage to nail
79167	Open fracture of distal phalanx of finger
45427355	Open fracture finger distal phalanx
133646	Injury of digital nerve
45450746	Digital nerve injury
45464147	Mallet finger with closed tendon injury
4016831	Mallet finger with closed tendon injury
45427476	Closed crush injury, finger
4051900	Closed crush injury, finger
45460638	Closed traumatic dislocation of finger not otherwise specified
4050226	Open wound of hand, palm
45434092	Open wound of hand, palm
45420958	Open wound of hand, excluding fingers, NOS
4144188	Cutting own wrists
45441449	Cutting own wrists
45420893	Closed fracture finger middle phalanx, base
4015879	Closed fracture finger middle phalanx, base
45477495	Closed fracture of phalanx or phalanges, unspecified
76542	Open fracture of one or more phalanges of hand
437123	Closed fracture of hamate bone of wrist
45477489	Closed fracture hamate
37116302	Superficial injury of elbow, forearm and wrist
45437427	Superficial injury of elbow, forearm and wrist
45480909	Wrist and hand sprain NOS
4019244	Rupture tendon forearm or wrist
45440685	Rupture tendon forearm or wrist
4309673	Hyperextension injury of finger
45474177	Hyperextension injury of finger
4050227	Open wound of hand with complication
45500740	Open wound of hand with complication
45434059	Rupture tendon of finger
45480876	Closed fracture finger metacarpal base

4010386	Closed fracture finger metacarpal base
4019248	Rupture of tendon of finger
4129405	Open wound of hand
45430771	Open wound of hand without mention of complication
45434090	Open wound of elbow, forearm and wrist
45434024	Dislocation of finger or thumb not otherwise specified
45457308	Wrist fracture - closed
4009459	Closed fracture finger proximal phalanx, base
45497328	Closed fracture finger proximal phalanx, base
4154164	Fingernail injury
45434170	Other fingernail injuries
74768	Closed fracture of triquetral bone of wrist
45487469	Closed fracture triquetral
45504139	Other finger injuries NOS
80223	Closed fracture finger middle phalanx
45517397	Closed fracture finger middle phalanx
45474250	Trapped finger
45494068	Closed fracture finger metacarpal
4015871	Closed fracture finger metacarpal
45420984	Superficial injury of hand, excluding fingers
45514152	Contusion, finger
4055866	Superficial injury of hand, excluding fingers
45440671	Fracture-dislocation/subluxation finger/thumb
4138281	Fracture subluxation of finger
4138275	Fracture dislocation of wrist joint
45437347	Fracture-dislocation or subluxation of wrist
45484138	Closed fracture of one or more phalanges of hand
4052671	Contusion, fingernail
45467573	Contusion, fingernail (includes subungual haematoma)
45441015	Self inflicted lacerations to wrist
45467460	Closed fracture finger distal phalanx
4297304	Closed fracture of distal phalanx of finger

45487474	Closed fracture finger metacarpal neck
4015354	Closed fracture finger metacarpal neck
45424110	Multiple fractures of fingers
4297437	Fracture of multiple sites of phalanges of hand
4319560	Self inflicted lacerations to wrist
45510677	Traumatic amputation of finger(s)
4086197	Superficial injury of hand
45510698	Superficial injury of hand NOS, without mention of infection
45460656	Tendon injury - hand
4319151	Traumatic amputation of finger
45450690	Superficial injury of wrist NOS
4164346	Superficial injury of wrist
45434023	Closed traumatic dislocation of finger, unspecified
45424221	Contusion wrist or hand
4134947	Tendon injury - hand
81186	Contusion of wrist
45420992	Contusion, wrist
4052668	Contusion wrist or hand
72465	Closed fracture of one or more phalanges of hand
45510600	Closed fracture finger proximal phalanx
45457420	Crush injury, wrist
75114	Crushing injury of wrist
443115	Closed fracture finger proximal phalanx
438268	Closed traumatic dislocation of joint of finger
45453937	Dislocation or subluxation of finger or thumb
45500769	Superficial of injury finger(s)
74211	Superficial injury of finger without infection
45427462	Superficial injury of finger NOS, without mention of infection
45460729	Foreign body in hand
4058346	Foreign body in hand
4166906	Superficial injury of finger
37311123	Dislocation of digit of hand

45434093	Open wound, finger
45497489	Finger injury
45497455	Contusion, finger, unspecified
45447320	Open wound of wrist, unspecified
45507383	Crush injury, hand, excluding fingers
4057476	Crush injury, hand, excluding fingers
45450626	Hand sprain
4170462	Foreign body - finger
45424213	Foreign body - finger
4052669	Contusion, hand, excluding finger
45520741	Contusion, hand, excluding finger
73045	Sprain of hand
4054062	Open wound of wrist
45497334	Fracture of bone of hand
4071876	Fracture of hand
45514084	Sprain of wrist and hand
45464313	[X]Unspecified injury of wrist and hand
73649	Contusion of finger
81169	Superficial foreign body of finger without major open wound AND without infection
45464215	Splinter of finger, without major open wound or mention of infection
4018956	Sprain of wrist and/or hand
45464257	Unspecified injury of hand
45450596	Fracture of one or more phalanges of hand NOS
45500665	Closed fracture of the scaphoid
80552	Closed fracture of scaphoid bone of wrist
45490765	Sprain finger
4134309	Sprain of ligament of finger
45517399	Fracture of other finger
45443714	Mallet finger
77647	Mallet finger
45487472	Fracture at wrist and hand level
4015350	Fracture at wrist and/or hand level

45494195	Crush injury, finger(s)
45427353	Fracture of one or more phalanges of hand
45454070	Unspecified injury of wrist
45520711	Open wound of finger(s) NOS
75406	Crushing injury of finger
4137945	Fracture of scaphoid bone of wrist
45507274	Fracture of scaphoid
45460780	Other finger injuries
45480948	Open wound of hand, excluding finger(s)
4056160	Open wound of hand, excluding finger(s)
4226282	Fracture of phalanx of hand
45447323	Open wound of finger(s) or thumb
45510599	Finger fracture
4057580	Fracture of phalanx of finger
4054063	Open wound of finger
45481020	Other hand injury, excluding finger
45447392	Other wrist injuries
45471043	Other finger injuries, unspecified
80004	Injury of hand
444129	Injury of wrist
81454	Injury of finger

Supplementary Material 1b. Read codes: hand trauma surgery cohort

Concept	Concept name
45505860	Amputation of finger NEC
4297321	Amputation of finger, except thumb
4263590	Amputation of hand
4078561	Amputation of phalanx of finger
45522551	Amputation of phalanx of finger
45445831	Closed (or no) reduction of fracture and external fixation
45522495	Closed (or no) reduction of fracture and internal fixation
45472707	Closed reduction # arm
4321394	Closed reduction of dislocation of finger
45522527	Closed reduction of dislocation of finger
4088717	Closed reduction of dislocation of wrist
45419494	Closed reduction of dislocation of wrist
45509329	Closed reduction of fracture
4313275	Closed reduction of fracture
4160364	Closed reduction of fracture and external fixation
45509189	Closed reduction of fracture dislocation of joint and internal fixation of joint
4106591	Closed reduction of fracture dislocation of joint and internal fixation of joint
45425937	Closed reduction of fracture of finger
4079111	Closed reduction of fracture of finger
45462593	Closed reduction of fracture of radius and or ulna
4079114	Closed reduction of fracture of radius and/or ulna
4075918	Closed reduction of fracture of small bone and fixation using screw
45472704	Closed reduction of fracture of small bone and fixation using screw
45515961	Closed reduction of fracture of thumb
4079112	Closed reduction of fracture of thumb
45502542	Closed reduction of fracture of upper limb
4146119	Closed reduction of fracture of upper limb
4076443	Closed reduction of fracture of wrist
45455815	Closed reduction of fracture of wrist
4188615	Closed reduction of fracture with internal fixation
4074406	Complex reconstruction operations on hand and foot
45452449	Complex reconstruction operations on hand and foot
45509175	Debridement of open fracture
4101851	Debridement of open fracture
4229905	Exploration of tendon
45476006	Exploration of tendon NEC
4078271	Finger operation

45439228	Finger operation
4173316	Fixation of tendon
45524661	Follow-up care involving plastic surgery
45459205	Hand operation
4003065	Hand reconstruction
45522492	K wiring of fracture
4343908	Ligament reconstruction
45429298	Manipulation of fracture of bone NEC
4308659	Manipulation of wrist joint
45492651	Manipulation of wrist joint
45505852	Manipulation of wrist joint under anaesthetic
45498859	Microsurgical graft to peripheral nerve NEC
45455463	Microsurgical repair of peripheral nerve
4066794	Microsurgical repair of peripheral nerve
45498861	Microsurgical repair of peripheral nerve NOS
4313570	Open reduction of fracture
4083670	Open reduction of fracture dislocation of joint
45512580	Open reduction of fracture dislocation of joint and fixation of joint, unspecified
4071354	Open reduction of fracture with internal fixation
45489340	Open surgical fracture of bone
4313023	Operation on tendon
4308878	Operation on tendon sheath
4010247	Operative procedure on hand
45476054	Other closed reduction of fracture of bone
45486006	Other closed reduction of fracture of bone NOS
45505811	Other complex reconstruction of hand
45439185	Other complex reconstruction of hand NOS
45419486	Other interposition reconstruction of joint
45476009	Other operation on tendon NOS
45429252	Other operations on sheath of tendon
45522457	Other operations on tendon
45419466	Other primary open reduction of fracture of bone
45465955	Other primary open reduction of fracture of bone NOS
45489371	Other reconstruction of joint
45519238	Other reconstruction of ligament
45442561	Other specified amputation of hand
45439157	Other specified operation on tendon
45459154	Other specified primary open reduction of fracture of bone and intramedullary fixation
45452416	Other specified primary repair of tendon
45465986	Other specified reconstruction of ligament
45465918	Plastic repair of tendon
4071370	Plastic repair of tendon

45429249	Plastic repair of tendon NOS
4042311	Plastic repair procedure
45425938	Primary closed reduction of fracture alone
45489346	Primary closed reduction of fracture and internal fixation with screw(s)
4076321	Primary closed reduction of fracture and internal fixation with screw(s)
45479386	Primary closed reduction of fracture and internal fixation with wire
45462587	Primary closed reduction of fracture and other internal fixation
45432554	Primary closed reduction of fracture and skeletal traction NEC
45482672	Primary closed reduction of fracture and wire fixation
4076317	Primary closed reduction of fracture and wire fixation
45442520	Primary external fixation of fracture
4077773	Primary external fixation of fracture
45482337	Primary microsurgical repair of peripheral nerve NEC
45515951	Primary open reduction of fracture alone
45505823	Primary open reduction of fracture and cast immobilisation
4077482	Primary open reduction of fracture and cast immobilization
45486001	Primary open reduction of fracture and external fixation
4076193	Primary open reduction of fracture and external fixation
4078965	Primary open reduction of fracture and internal fixation with K-wire
45425934	Primary open reduction of fracture and internal fixation with K-wire
4182345	Primary open reduction of fracture and internal fixation with plate
45472703	Primary open reduction of fracture and internal fixation with plate NEC
45495884	Primary open reduction of fracture and internal fixation with screw(s)
4078967	Primary open reduction of fracture and internal fixation with screw(s)
4075782	Primary open reduction of fracture and internal fixation with tension band wiring
45495883	Primary open reduction of fracture and internal fixation with tension band wiring
45492617	Primary open reduction of fracture and intramedullary nail fixation
45455809	Primary open reduction of fracture and other internal(extramedullary) fixation
4116617	Primary open reduction of fracture dislocation
4077794	Primary open reduction of fracture dislocation and fixation with plate(s)
45492640	Primary open reduction of fracture dislocation and fixation with plate(s)
4106584	Primary open reduction of fracture dislocation and wire fixation
45522525	Primary open reduction of fracture dislocation and wire fixation
45479407	Primary open reduction of fracture dislocation of joint and combined internal and external fixation
45462615	Primary open reduction of fracture dislocation of joint NEC
4324896	Primary open reduction of fracture dislocation of joint with combined internal and external fixation
45492618	Primary open reduction of fracture of bone and external fixation
45519206	Primary open reduction of fracture of bone and extramedullary fixation
4075780	Primary open reduction of fracture of bone and extramedullary fixation
45435920	Primary open reduction of fracture of bone and extramedullary fixation NOS
45472700	Primary open reduction of fracture of bone and intramedullary fixation
4074392	Primary open reduction of fracture of bone and intramedullary fixation

45445826	Primary open reduction of fracture of bone and intramedullary fixation NOS
45509163	Primary open reduction of fracture of long bone and complex extramedullary fixation NEC
45479384	Primary open reduction of fracture of long bone and fixation using rigid nail NEC
4078961	Primary open reduction of fracture of small bone and fixation using screw
45425933	Primary open reduction of fracture of small bone and fixation using screw
45449059	Primary plastic repair tendon
4071371	Primary plastic repair tendon
45442476	Primary repair of tendon
4071373	Primary repair of tendon
45515905	Primary repair of tendon NOS
45442477	Primary repair of tendon using graft
4072044	Primary repair of tendon using graft
45425892	Primary repair of tendon using tendon transfer procedure
4071374	Primary repair of tendon using tendon transfer procedure
45449060	Primary simple repair of tendon
4072045	Primary simple repair of tendon
45495888	Primary wire fixation of fracture
4077626	Primary wire fixation of fracture
4118094	Reattachment of finger
4321095	Reconstruction of joint
45422706	Reconstruction of ligament NOS
45452466	Remanipulation of fracture of bone NEC
4311041	Repair of artery
45492471	Repair of other artery
45512403	Repair of other artery NOS
45478249	Repair of scarred tissue
4121003	Repair of single tendon
45449158	Replantation of finger NEC
45509165	Secondary open reduction of fracture of bone and intramedullary fixation however further qualified
4300388	Surgical manipulation of wrist joint
45469381	Suture of tendon
45432505	Tendon operations
45452419	Tendon operations NOS
4072035	Tendon transfer to extensor tendon of hand
45429246	Tendon transfer to extensor tendon of hand
45425890	Tenodesis
45486045	Terminalisation of finger
4106037	Terminalization of finger

Supplementary Material 1a. Antibiotics, NSAIDs and opioid analgesics

All antibiotics		
Floxacillin	Norfloxacin	Ciprofloxacin
Amoxicillin	Polymyxin B	Minocycline
Clavulanate	Cefuroxime	Gentamicin
Erythromycin	Cefaclor	Nitrofurantoin
Fusidate	Oxytetracycline	Cephradine
Clarithromycin	Clindamycin	Ampicillin
Trimethoprim	Cephalexin	Azithromycin
Neomycin	Doxycycline	Sulfamethoxazole
Metronidazole	Lymecycline	Tetracycline
Penicillin V	Chloramphenicol	Cefixime

SSI antibiotics
Floxacillin
Amoxicillin
Clavulanate
Erythromycin
Clarithromycin
Clindamycin
Doxycycline
Ciprofloxacin
Chloramphenicol

NSAIDs
Ibuprofen
Diclofenac
Naproxen

Opiates
Codeine
Dihydrocodeine
Tramadol

Supplementary Material 3. Demographic data for CPRD cohorts

	Hand trauma cohort	95%CI	Hand trauma surgery cohort	95%CI
**Total n**	641,223	–	3,088	–
**Sex: Female (%)**	273,920 (42.7)	–	1068 (34.6)	–
**Sex: Male (%)**	367,303 (57.2)	–	2020 (65.4)	–
**Mean age, years (SD)**	34.9 (22.2)	34.9 – 35.0	39.2 (21.5)	38.4 – 40.0
